# Mediating effect of meaning in life on death anxiety and attitude toward palliative care among undergraduate nursing students

**DOI:** 10.1186/s12904-024-01472-w

**Published:** 2024-06-05

**Authors:** Gui-Ru Xu, Wen-Ying Yu

**Affiliations:** School of Nursing, Fujian Medical University, No. 1, Xuefu North Road, Minhou County, Fuzhou City, 350122 Fujian Province China

**Keywords:** Death anxiety, Mediating effect, Undergraduate nursing students, Palliative care, Presence of meaning in life

## Abstract

**Background:**

This study investigates the mediating effect of meaning in life between death anxiety and attitude toward palliative care among nursing students.

**Methods:**

We enrolled 363 undergraduate nursing students using a convenience sampling method as the respondents and conducted a survey using general information about nursing students, the Chinese version of the FATCOD-B Scale, the Chinese version of the Death Anxiety Scale, and the Chinese version of the Meaning in Life Questionnaire. The SPSS25.0 statistical software was used to analyze the mediating effect.

**Results:**

The mean total attitude score toward palliative care was (104.72 ± 10.62). Death anxiety had a significant negative predictive effect on the attitude toward palliative care (β = -0.520, *P* < 0.01). When the mediating variable of the presence of meaning in life was included, the negative predictive effect of death anxiety on attitude toward palliative care remained significant (β = -0.379, *P* = 0.036); the mediating effect (-0.141) accounted for 27.12% of the total impact (-0.520).

**Conclusions:**

The presence of meaning in life mediates the relationship between death anxiety and attitude toward palliative care. This implies that nursing educators, through their role in educating nursing students about the meaning of life, can significantly influence the development of a positive attitude toward palliative care.

## Background

Palliative care refers to the comprehensive care provided by a multidisciplinary team to patients who are living with a terminal or chronic illness and their family members, including physical, psychological, spiritual, and social support, to help patients die comfortably, peacefully and with dignity [1]. The National Health and Family Planning Commission issued the *Basic Standards and Management Regulations for Palliative Care Centers (Trial)* and the *Palliative Care Practice Guidelines (Trial)* on February 9, 2017, emphasizing the national significance of palliative care [2]. However, palliative care in China is still in its infancy [3]. It was reported that clinical nurses who feel emotionally unprepared to care for dying patients may become upset and share their worries or relay their concerns to patients and families [4]. As future nurses, nursing students should be educated and prepared to offer palliative care. The Chinese Ministry of Education’s National Standard of Teaching Quality for Nursing Students also suggested that nursing students become acquainted with palliative care [5]. Currently, there is no unified teaching curriculum for palliative care in China, and most nursing training programs in colleges do not include specialized courses on palliative care [6]. As a result, the need for ongoing, evidence-based nurse education on the concepts and aims of palliative care is critical [7]. 

Nurses should have sufficient knowledge, practice, and a positive attitude to provide high-quality, effective palliative care [8]. Previous evidence has revealed that the attitudes of nursing students towards death and palliative care were influenced by university type, academic level, and gender [5]. Understanding nursing students’ attitudes regarding palliative care should be prioritized before developing a curriculum focused on palliative care. Numerous studies have found that nursing students’ attitudes regarding palliative care were associated with various characteristics, including cultural background, experience caring for patients, academic parameters, knowledge and education about palliative care, religious belief, death attitude, and meaning in life [9–16]. 

Death anxiety (DA) is defined as a ‘negative emotional reaction induced by the anticipation of a condition in which the self does not exist’ [17], followed by feelings of fear or dread [18]. It is claimed that one reason for dread may be the ‘unknowable’--what occurs after death [19]. DA has been investigated as a possible factor impacting the attitude toward palliative care [20]. Thiemann et al. discovered that medical students’ DA was moderate and that higher levels of DA were associated with increased concern about delivering palliative care [19]. Peters L et al. and Brockopp DY et al. also claimed that nursing staff who have a high level of DA may reduce communication with patients who are living with a terminal or chronic illness and display a negative attitude toward palliative care [21, 22]. Although studies concluded the DA had effects on the attitude toward palliative care, the specific mechanism has not been fully explored, especially in China, where denial of death appears to be more widespread.

Meaning in Life refers to the individual’s integrated perspective of the meaning and purpose of self-existence [23]. Empirical research shows a link between meaning in life and attitudes toward palliative care [24]. Ensuring that nurses grasp the significance of meaning in life when caring for individuals with terminal or chronic illnesses is particularly vital, especially considering the emotional distress they may encounter [25, 26]. In addition, Çakar et al. found a negative relationship between death anxiety and meaning in life [27]. According to terror management theory, a social and evolutionary psychology theory initially proposed by Jeff Greenberg, Sheldon Solomon, and Tom Pyszczynski, humans require control over detrimental psyches caused by death events based on an instinct for self-preservation, such as meaning in life. In theory, meaning in life may reduce the influence of death dread on attitudes toward palliative care [9, 28]. 

Nursing students’ death anxiety and meaning in life are particular indications that can influence their attitude toward palliative care. Evaluating these indicators is critical for understanding the current status of palliative care education in nursing and contributing to developing palliative care-related courses.

Nursing students’ DA and meaning in life are particular indications that can influence their attitude toward palliative care. Based on literature reviews, the current study developed a mediating model among a sample of Chinese nursing students to assess the link between death anxiety, life meaning, and attitudes toward palliative care.

## Methods

###  Design

A mediating model was developed to assess the link between death anxiety, life meaning, and attitudes toward palliative care. In this investigation, the following assumptions were established: (1) Death anxiety and meaning in life are linked to attitudes toward palliative care; (2) Meaning in life acts as a mediator between death anxiety and attitudes toward palliative care.

###  Participants

From October 2021 to April 2022, 363 nursing students from 7 Class A tertiary hospitals in Fujian Province were selected using a convenience sampling method to participate as respondents. These students are all from undergraduate nursing schools, receiving three years of on-site instruction and at least ten months of continuous clinical practice instruction. Inclusion criteria: (1) ≥ 18 years old; (2) fourth-year undergraduate nursing students; (3) experiences clinical internship; (4) agree to participate in this study. Exclusion criteria: (1) refuse to accept the URL link or QR of the questionnaire; (2) individuals with clinically diagnosed mental illnesses, whether they are under medication or not. The participants were reminded of the voluntary nature of their participation in the study and their autonomy to withdraw at any time before the analysis commenced. The survey was anonymous.

###  Study measures

(1) General information questionnaire. We compiled this questionnaire to include questions on gender, age, religion, whether a relative died in the past year, whether there is currently a seriously ill family member, discussions about death at home, and previous palliative care education.

(2) The Meaning in Life Questionnaire (MLQ), developed by Steger et al. [29]. and revised by Wang Xinqiang [30], is a widely used measurement tool and is divided into two sub-scales: the presence of meaning in life (MLQ-P) and the search for meaning in life (MLQ-S). Each sub-scale has five questions and adopts a Likert 7-point scoring method, from 1 “Absolutely untrue” to 7 “Absolutely true”; the internal consistency α coefficients of the overall scale and MLQ-S and MLQ-P scales were 0.802, 0.825, and 0.760, respectively, indicating that the scale and its dimensions are reliable. The correlation coefficient between MLQ-S and MLQ-P was 0.307, and their correlation coefficients with the overall scale were 0.822 and 0.795, respectively, with *p* < 0.001, indicating that the scale has good construct validity and can be used in this study.

(3) Death Anxiety Questionnaire. Professor Templer developed the Death Anxiety Scale (T-DAS) in the USA, and Yang Hong developed the Chinese version of T-DAS [31] based on a cross-cultural adaptation. There are 15 entries covering four dimensions: emotion, stress and distress, time awareness, and cognition. The scale is scored on a “Yes/No” basis, with each entry scored 0/1; the total score ranges from 0 to 15, with a total score > 7 indicating high death anxiety, and the higher the score, the more severe the death anxiety. The total Cronbach’s α coefficient of the scale was 0.71, indicating that it is reliable and valid.

(4) The Frommelt Attitude Toward Care of Dying (FATCOD) scale was developed by Frommelt in 1991 to assess the attitude of nurses toward caring for patients who are terminally ill and their families [32]; it is the most widely used scale globally for such an assessment. Researchers in China revised the scale through a rigorous process of cross-cultural adaptation, confirmed the reliability and good applicability of the Chinese version of the FATCOD by analyzing its reliability and validity, and ensured the scale’s content validity [33]. The scale consists of 30 items and employs a Likert 5-point scoring method, ranging from “Strongly disagree” to “Strongly agree,” with positive questions scored from 1 to 5 and negative questions scored from 5 to 1. The total score on the scale ranges from 30 to 150, and the higher the score, the more positive the attitude of the respondents toward caring for patients who are terminally ill. After the normality test, the total score of the scale conformed to a normal distribution, and the internal consistency reliability was described by Cronbach’s α reliability coefficient, α = 0.805; the split-half reliability was 0.697, *P* < 0.001; and the two indexes indicated that the Chinese version of the scale had good reliability and could be used in this study.

###  Survey methods

The current questionnaire-based survey was conducted using the social media platform WeChat and the online professional survey platform “Wenjuanxing” (www.wjx.cn). First, the research assistant enters the questionnaire into the “Wenjuanxing” platform, which generates a survey webpage or QR code. The research assistant then chose 5–10 students for a pilot survey to ensure the viability of the study. Finally, the research assistant used snowballing to recruit nursing interns from seven hospitals and circulate questionnaire websites or QR codes via WeChat. To ensure the completeness of the questionnaire, we set the “Answer all questions before submitting” option in the platform. After collecting the questionnaire, the research assistant checked the possibilities and eliminated invalid questionnaires, such as all answers being the same or with regularity. Before distributing the questionnaire, unified instructions were provided to fully inform the respondents of the study’s purpose, significance, questionnaire filling method, and precautions and to inform them that the questionnaire’s content was for study purposes only.

Kendall’s clinical research sample size estimation formula states that the sample size should be 5 to 10 times the number of variables [34]. Taking 10% invalid questionnaires into account, the sample size determined in this study is 342. Finally, 400 questionnaires were distributed, and 363 valid questionnaires were returned, with an effective recovery rate of 90.75%. The duration for the participants to complete the questionnaire varied between 10 and 30 min.

###  Statistical methods

SPSS 25.0 statistical software was used for statistical analysis. The mean and standard deviation are used to describe the enumeration data. Pearson’s correlation analysis was used to investigate the correlation between the presence of meaning in life, death anxiety, and attitude toward palliative care of nursing students.

The bootstrapping method is a nonparametric method for indirect effects, and its confidence intervals (CI) can better explain the irregularity of the indirect effect’s sampling distribution [35]. Therefore, a bootstrapping-based method was used to test the present study’s mediating effects. Our study generated 5000 bootstrap samples by PROCESS macro plug-in in SPSS 25.0 to produce bootstrap CI for the indirect effect. At an alpha level of 0.05, an indirect impact is considered significant if its 95% CI does not include zero.

## Results

###  Basic information of respondents

Among the 363 nursing students, 85.95% (312) were female; 52.07% (189) were aged 22 to 23 years; 72.45% (263) had their registered residence in rural areas; 92.56% (336) had no religion; 85.40% (310) experienced professional internship more than six months; 74.93% (272) had no family member who died in the past year; 92.01% (334) did not have a relative who was receiving palliative care or receiving end of life care or terminally ill; 54.55% (198) avoided discussing death at home; and 52.62% (191) had not taken palliative care-related courses in the past but had learned palliative care-related content in other classes. See details in Table 1.

**Table 1:** General information of the respondents [*n* = 363, respondents (percentage, %)

Item	Number of respondents	Percentage (%)
Gender		
Female	312	85.95
Male	51	14.05
Age (years)		
18–19	3	0.83
20–21	162	44.63
22–23	189	52.07
24∼	9	2.48
Registered permanent residence		
Urban	100	27.55
Rural	263	72.45
Religion		
Yes	27	7.44
No	336	92.56
Duration of internship (months)		
4	22	6.06
5	31	8.54
6	89	24.52
7	106	29.20
8	115	31.68
Have you experienced the death of a relative in the past year		
Unwilling to answer this question	22	6.06
Yes	69	19.01
No	272	74.93
Currently, is there any seriously ill patient at your home		
Yes	29	7.99
No	334	92.01
Family talks about death		
Very openly	131	36.09
Try to avoid talking about death	198	54.55
Never talk about death	34	9.37
Previous palliative care education received		
Have taken specialized course about palliative care and death	136	37.47
Have not taken related course, but have learnt related content in other courses	191	52.62
Never studied about palliative care and death	36	9.92

###  Scores of meaning in life, death anxiety, and attitude toward palliative care of nursing students

The mean total score of attitude toward palliative care was (104.72 ± 10.62); the mean total score of death anxiety was (7.83 ± 2.90); and the mean total score of meaning in life was (50.49 ± 8.66). The total score of each scale and the mean score of each dimension are shown in Table 2.

**Table 2:** Scores of nursing students on each scale

Scale	Score (mean ± SD)	Scale	Score (mean ± SD)
Meaning in life		Death anxiety	
Total score	50.49 ± 8.66	Total score	7.83 ± 2.90
Presence of meaning in life	23.93 ± 5.08	Emotion	2.18 ± 1.44
Search for meaning in life	26.56 ± 5.27	Stress and pain	2.64 ± 1.14
Attitude toward palliative care		Time awareness	0.92 ± 0.76
Total score	104.72 ± 10.62	Cognition	1.44 ± 0.74
Positive attitude toward caring for terminally ill patients	51.29 ± 7.21		
Patient- and family centric nursing awareness	53.43 ± 6.26		

###  Correlation analysis of meaning in life, death anxiety and attitude toward palliative care of nursing students

Death anxiety was negatively correlated with attitude toward palliative care (*r* = -0.142, *P* = 0.007); death anxiety was not correlated with the meaning in life and the search for meaning in life but was negatively correlated with the presence of meaning in life (*r* = -0.110, *P* = 0.037); and the attitude toward palliative care was positively correlated with the meaning in life, the search for meaning in life, and the presence of meaning in life. See details in Table 3.

**Table 3:** Correlation analysis of death anxiety, attitude toward palliative care, and meaning in life (r, *n* = 363)

Item	Total score of meaning in life	Search for meaning in life	Presence of meaning in life
Death anxiety	-0.023	0.067	-0.110^a^
Attitude toward palliative care	0.421^b^	0.344^b^	0.362^b^

^a^: *P* < 0.05; ^b^: *P* < 0.01

###  Mediating effects of the presence of meaning in life on death anxiety and attitude toward palliative care of nursing students

The mediating effect test was conducted with death anxiety as the independent variable, meaning in life as the mediating variable, and attitude toward palliative care as the dependent variable. The upper and lower 95% CI of the direct effect of death anxiety on the attitude toward palliative care and the mediating effect of the presence of meaning in life (Bootstrap 5000 times) did not contain 0, indicating that death anxiety can not only directly predict the attitude toward palliative care, but also predict the attitude toward palliative care through the mediating effect of the presence of meaning in life. The direct effect (-0.379) and mediating effect (-0.141) accounted for 72.88% and 27.12% of the total impact (-0.520), respectively. See details in Table 4.

**Table 4:** Mediating effect analysis of presence of meaning in life among nursing students

	Point estimate	Bootstrap standard error	*P*	Bootstrapping 95% CI	
				lower	upper
Total effect	-0.520	0.191	< 0.05	-0.895	-0.145
Direct effect	-0.379	0.180	< 0.05	-0.733	-0.025
Mediating effect	-0.141	0.073	< 0.05	-0.294	-0.005

## Discussion

This study examined the relationship between death anxiety, meaning in life, and attitudes toward palliative care among Chinese undergraduate nursing students in their final year internship. Nursing students participating in the study expressed a moderate positive attitude toward palliative care. Overall, the study investigated a mediating hypothesis, and the findings demonstrated that death anxiety was strongly and negatively connected with attitude toward palliative care and that this correlation was mediated by meaning in life.

The results of this study revealed that the score of attitudes toward palliative care of nursing students was (104.72 ± 10.62), accounting for approximately 69% of the total score. This score is slightly higher than the third-year nursing students who already had some practical experience in a hospital (101.34 ± 7.75) [36] and the two or third-year nursing students without experience in an integrated clinical internship in China (98.08 ± 8.02) [9]. It could be because most nursing students in our study had a 6 to 8-month internship. According to Liu Yang’s research [37], nursing students with a 7-month internship term had comparatively positive attitudes toward palliative care, maybe because they had more clinical exposure. Nursing students encountering the phenomena of death in practical settings may influence their favorable views toward dying [38]. It is worth mentioning that only 37.47% of participants had attended specific palliative care and death-related courses, which may explain why the students in this study exhibited a more negative attitude toward palliative care than students studied by Dimoula et al. [11] and Fristedt S et al. [39]. As a result, palliative care education and training should be developed in nursing schools. A good attitude toward palliative care can be improved by taking a palliative care-related curriculum, ensuring students’ theoretical and practical skills, and with hands-on, mandatory training experiences in this specialized care setting [9, 40].

The current study found that nursing students’ death anxiety correlated negatively with their attitudes toward palliative care [41]. According to terror management theory, the fear of death was closely tied to attitudes toward palliative care. Furthermore, cognitive behavior therapy theory may clarify the relationship between the two factors, which claim that emotion (feelings/mood), cognitions (thoughts/attitudes), and behaviors (actions/physical reactions) all influence each other [41]. The clinical experience of caring for patients who are dying and those who have died may make nursing students aware of their mortality and provoke emotional reactions that are determined by individuals’ attitudes.

Conversely, our findings demonstrated that nursing students’ presence of meaning in life was positively related to their attitudes toward palliative care, similar to previous studies. A study conducted by Sim in Korea found that meaning in life significantly predicted nursing students’ attitudes toward palliative care [42]. Another investigation carried out by Miller et al. on 277 students enrolled in a massive open online course concerning death in Australia also identified the strongest correlation between the presence of meaning in life and an increased capacity to cope with palliative care [43]. The presence of meaning in life represents the cognitive part of meaning in life and refers to comprehension and acknowledgment of one’s life’s purposes, goals, or missions; this shows the extent to which individuals believe their lives are meaningful. According to Steger et al., a higher presence of meaning in life is helpful for adaptive resources, overall psychological well-being, and pleasant effects [44], which may contribute to students’ effective coping with death events and positive attitudes toward palliative care.

The present study found a statistically negative relationship between death anxiety and the presence of meaning in life. This result was inconsistent with previous research; more research is needed to verify the relationship between death anxiety and the meaning of life. Existentialism contends that death dread raises people’s awareness of their finite lives and encourages them to find meaning in their lives [44–46]. However, participants in our study were students who may be more prone to suffer from stress and distress when facing death scenarios compared to experienced nurses. This leads them into a chaotic state about their existence, value, and mission, and thus may be unable to perceive their life meaning. Meanwhile, the presence of meaning in life showed a mediated effect between death anxiety and attitude toward palliative care. Nursing students with a stronger sense of meaning in life have a more positive attitude toward death, which can stimulate their positive understanding of death and help alleviate their death anxiety.

## Conclusion

Nursing students in the current study had fewer positive attitudes toward palliative care than students in Western countries. These findings suggested that palliative care education should be integrated into the nursing curriculum in China and that nursing educators should pay special attention to strengthening nursing students’ attitudes toward dealing with the psychological challenges of palliative care. Because the presence of meaning in life could moderate the association between death anxiety and attitude toward palliative care, nursing educators might assist nursing students in acquiring a good attitude toward palliative care by educating them about meaning in life.

### Limitation

This study has several limitations. To begin with, the convenience sample of nursing students collected may have accurately represented only some nursing students in mainland China. There is diversity in the content of palliative care lectures or courses within nursing institutions, which may contribute to student perceptions that differ from those of our sample. Second, this cross-sectional study only captured students’ perceptions of palliative care within a particular academic year (fourth). As a result, we cannot comment on attitude changes as students proceed through their education. Finally, because self-report instruments were used, the respondents’ opinions and interests may impact the dependability of the data acquired.

### Implication

This study has implications in several aspects. First, because nursing students’ attitudes and education on death-related issues in China are still in their early stages, more research on the relationship between death anxiety and attitudes toward palliative care based on cultural background is required. Second, as palliative care education was lacking, comprehensive education and training programs should be developed to provide Chinese nursing students with the necessary information, abilities, and attitudes to cope with palliative care. Third, the presence of meaning in life has a positive effect on attitudes toward palliative care, as well as a suppressing impact on attitudes toward palliative care from death anxiety. As a result, designing, developing, and implementing sensitive interventions and training may help strengthen the Chinese nursing students’ presence of meaning in life and ultimately improve their attitudes towards palliative care.

## Data Availability

The datasets used and/or analyzed during the current study are available from the corresponding author upon reasonable request.
